# Safety of magnetic resonance imaging of patients with a new Medtronic EnRhythm MRI SureScan pacing system: clinical study design

**DOI:** 10.1186/1745-6215-9-68

**Published:** 2008-12-02

**Authors:** Richard Sutton, Emanuel Kanal, Bruce L Wilkoff, David Bello, Roger Luechinger, Inge Jenniskens, Michael Hull, Torsten Sommer

**Affiliations:** 1 St Mary's Hospital, Praed Street, W2 INY, London, UK; 2 UPMC Presbyterian, 200 Lothrop Street, Room D-132, Pittsburgh, PA 15213, US; 3 Cleveland Clinic 9500 Euclid Ave, Cleveland, OH 44195, US; 4 Orlando Regional Medical Center and Mid-Florida Cardiology Specialists, 1717 S. Orange Ave. Suite 105 Orlando, FL 32806, US; 5 Institute for Biomedical Engineering, University and ETH, Gloriastrasse 35, 8092 Zurich, Switzerland; 6 Medtronic, Bakken Research Center, Endepolsdomein 5, 6229 GW Maastricht, The Netherlands; 7 Medtronic, Inc., 7000 Central Ave. NE, Minneapolis, MN 55432-3576, US; 8 Department of Radiology, University of Bonn, Sigmund-Freud-Straße 25, 53127 Bonn, Germany

## Abstract

**Background:**

Magnetic Resonance Imaging (MRI) of patients with implanted cardiac devices is currently considered hazardous due to potential for electromagnetic interference to the patient and pacemaker system. With approximately 60 million MRI scans performed worldwide per year, an estimated majority of pacemaker patients may develop an indication for an MRI during the lifetime of their pacemakers, suggesting that safe use of pacemakers in the MRI environment would be clinically valuable. A new pacing system (Medtronic EnRhythm MRI™ SureScan™ and CapSureFix MRI™ leads) has been designed and pre-clinically tested for safe use in the MRI environment. The EnRhythm MRI study is designed to confirm the safety and efficacy of this new pacing system.

**Methods:**

The EnRhythm MRI study is a prospective, randomized controlled, unblinded clinical trial to confirm the safety and efficacy of MRI at 1.5 Tesla in patients implanted with a specifically designed pacemaker and lead system. The patients have standard indications for dual chamber pacemaker implantation. Successfully implanted patients are randomized in a 2:1 ratio to undergo MRI (MRI group) or to have no MRI scan (control group) at 9–12 weeks after pacemaker system implantation. Magnetic resonance (MR) scanning includes 14 head and lumbar scan sequences representing clinically relevant scans while maximizing the gradient slew rate up to 200 T/m/s, and/or the transmitted radiofrequency (RF) power up to SAR (specific absorption rate) levels of 2 W/kg body weight (upper limit of normal operating mode). Full interrogation of all device information and sensing and capture function are measured at device implantation, every follow-up and before and immediately after MRI in the MRI group and at the same time points in the control group. Complete pacemaker and lead evaluations are also done at one week and one month after the scan for the MRI and control group patients.

The primary endpoint is safe and successful completion of the MRI scan as measured by freedom from both MRI-procedure related complications and clinically significant changes in the sensing and capture function of the leads.

**Results:**

Results will be communicated after approximately 156 and 470 patients have completed 4 months of follow-up.

**Trial Registration:**

ClinicalTrials.gov identifier: NCT00433654.

## Background

Magnetic Resonance Imaging (MRI) of patients with pacemakers and other implanted devices is presently contraindicated, because of the potential hazards of this diagnostic method on active implants [1]. However, there is an increased need for MRI in these and other older patients [2]. With approximately 60 million MRI scans performed worldwide per year, an estimated majority of pacemaker patients may be indicated for an MRI during the lifetime of their pacemaker, suggesting that safe use of pacemakers in an MRI environment would be clinically valuable.

Several studies report that a small number of pacemaker patients underwent MRI scanning under controlled situations and by taking certain precautions. This was only done if the risk-benefit ratio was considered acceptable. Still, scanning pacemaker patients in an MRI is not without risk. Six non pacemaker-dependent patients died in Germany during an MRI examination that took place without any monitoring. In three of these cases, there was suggestive evidence that death may have been due to induced ventricular fibrillation [3]. Other studies have shown that pacing thresholds increased significantly from pre- to post-MRI examination [4,5]. Furthermore, animal testing has demonstrated that the temperature at the lead tip increased up to 20°C during MRI scanning of the heart [6], which could result in tissue damage.

Pacemaker systems can be designed to limit the potential risks of MRI for patients. Such a design has been realised, prompting a clinical trial to test its function with and without MRI. Since all available pacemaker systems are currently contraindicated for use during MRI, the new pacing system consisting of the EnRhythm MRI™ SureScan™ pacemaker and CapSureFix MRI™ Model 5086 leads are evaluated for safe use in the MRI environment. This pacing system is a modified version of the commercially available EnRhythm^® ^pacemaker and CapSureFix^® ^Novus Model 5076 leads. The design of the study evaluating this pacemaker system is reported in this paper.

Pacemaker design enhancements were made to minimize the energy induced on leads and discharged at electrodes. Hardware changes were made to ensure reliable operation while MRI is active. Leads were altered to reduce lead tip heating from radiofrequency (RF). Radiopaque labels are present on the lead and device to indicate that a system is implanted that can be used in the MRI. Furthermore, the pacemaker can be programmed to the MRI SureScan mode to permit appropriate function during MRI scanning. In this mode, the physician can choose either to programme an asynchronous pacing mode or a non-pacing mode. Asynchronous pacing will maintain appropriate pacing support throughout the MRI examination regardless of the noise induced on the pacing system. For those patients that are non-pacemaker dependent, the non-pacing mode is available. Diagnostic data collection, automatic system monitoring measurements, atrial arrhythmia detection and atrial arrhythmia therapy are suspended during MRI SureScan mode operation. Furthermore, before programming the MRI mode, the system reinforces the physician to check carefully the safety requirements necessary for the pacemaker patient to receive an MRI examination.

## Study design

This study is a prospective, randomized controlled, unblinded, multi-center investigational trial and is being conducted in approximately 75 centers in the United States (US), Canada, Europe and Middle East. After implantation of the Medtronic EnRhythm MRI SureScan system (Medtronic EnRhythm MRI™ SureScan™ pacemaker and Medtronic CapSureFix MRI™ leads), patients are required to have follow-up visits at 2 months, 2.5 months (between 9–12 weeks post-implant an MRI scan or no-MRI scan is performed, depending on randomization), 3 months (1 week post- MRI/no-MRI) and 4 months post-implant (1 month post-MRI scan/no-MRI). Further follow-up is required at 6 months post-implant and every 6 months thereafter until the study ends. Full interrogation of all device information and impedance, sensing and capture function are measured at implant, every follow-up and immediately before and after MRI in the MRI group and at the same time points in the control group. The system must be implanted for more than 6 weeks to allow the Implantable Pulse Generator (IPG) and leads to develop stable pacing and sensing function in the patient prior to allowing the patient to undergo an MRI scan.

A concurrent control group design was selected because the effect of MRI on capture and sensing measurements is unclear. The observed effect in this study will theoretically result from the sum of the natural variation in capture and sensing parameters common to all patients, added to the effect of the MRI examination. Without a randomized control group, it is impossible to isolate the effect of the MRI from the component common to all patients with an IPG system. The possible difference in the electrical measurements due to the MRI scan is precisely what study investigators wish to understand.

### Inclusion/exclusion criteria

The following in-/exclusion criteria are used. Criteria were primarily chosen to comply with the labeling of the pacemaker system.

#### Inclusion Criteria

• Patients who have a Class I or II indication for implantation of a dual chamber pacemaker according to the American College of Cardiology (ACC)/American Heart Association (AHA)/Heart Rhythm Society (HRS) guidelines [7].

• Patients must be able to undergo pectoral implantation.

• Patients who are able and willing to undergo elective MRI scanning without sedation (anxiolysis permitted).

• Patients who are geographically stable and available for follow-up at the study center for the length of the study.

#### Exclusion Criteria

• Patients who require a legally authorized representative to obtain consent.

• Patients with a mechanical tricuspid heart valve

• Patients with tricuspid valve disease including valve replacement.

• Patients for whom a single dose of 1.0 mg Dexamethasone acetate may be contraindicated.

• Patients who have a previously implanted pacemaker or an implanted cardioverter/defibrillator. Those who have abandoned pacemaker/ICD leads are excluded; however, patients with complete system explantation may be included.

• Patients who require an ICD rather than a pacemaker.

• Patients currently indicated, or for whom an indication is anticipated, to undergo another MRI procedure other than those specifically described in the study during the period of follow-up required by the study.

• Patients with previously implanted active medical devices (other than those stated above).

• Patients with non-MRI compatible devices (those above and others such as neurostimulators) or implanted material (e.g. non-MRI compatible sternal wires, biostimulators, metals or alloys).

• Patients with medical conditions that preclude the testing required by the protocol or limit study participation.

• Patients who are enrolled in or intend to participate in another clinical trial (of an investigational drug or device, new indication for an approved drug or device, or requirement of additional testing beyond standard clinical practice) during this clinical trial.

• Pregnant women, or women of childbearing potential and who are not on a reliable form of birth control.

• Patients with exclusion criteria required by local law (e.g age, breast feeding).

### Pacemaker implantation and measurements

The devices to be implanted are the model 5086 MRI CapSureFix^® ^active fixation leads for both right atrium and right ventricle. Typically right or left pectoral approaches via the subclavian, axillary or cephalic veins are used to introduce the leads, which are then advanced to the investigators' choice of location in the heart to sense and capture the atrial and ventricular myocardium. The EnRhythm MRI SureScan IPG is connected to the leads in the pectoral site.

After implantation, measurements of voltage stimulation thresholds at 0.2 and 0.5 ms pulse duration, impedance and sensing amplitudes measurements are taken in both the atrium and ventricle. The investigator completes a questionnaire concerning handling characteristics of the leads during implantation.

Measurements at the time of MR scanning are described below. Further sets of measurements of pacemaker function are made at every required follow-up visit.

### Randomization

**Figure 1:**
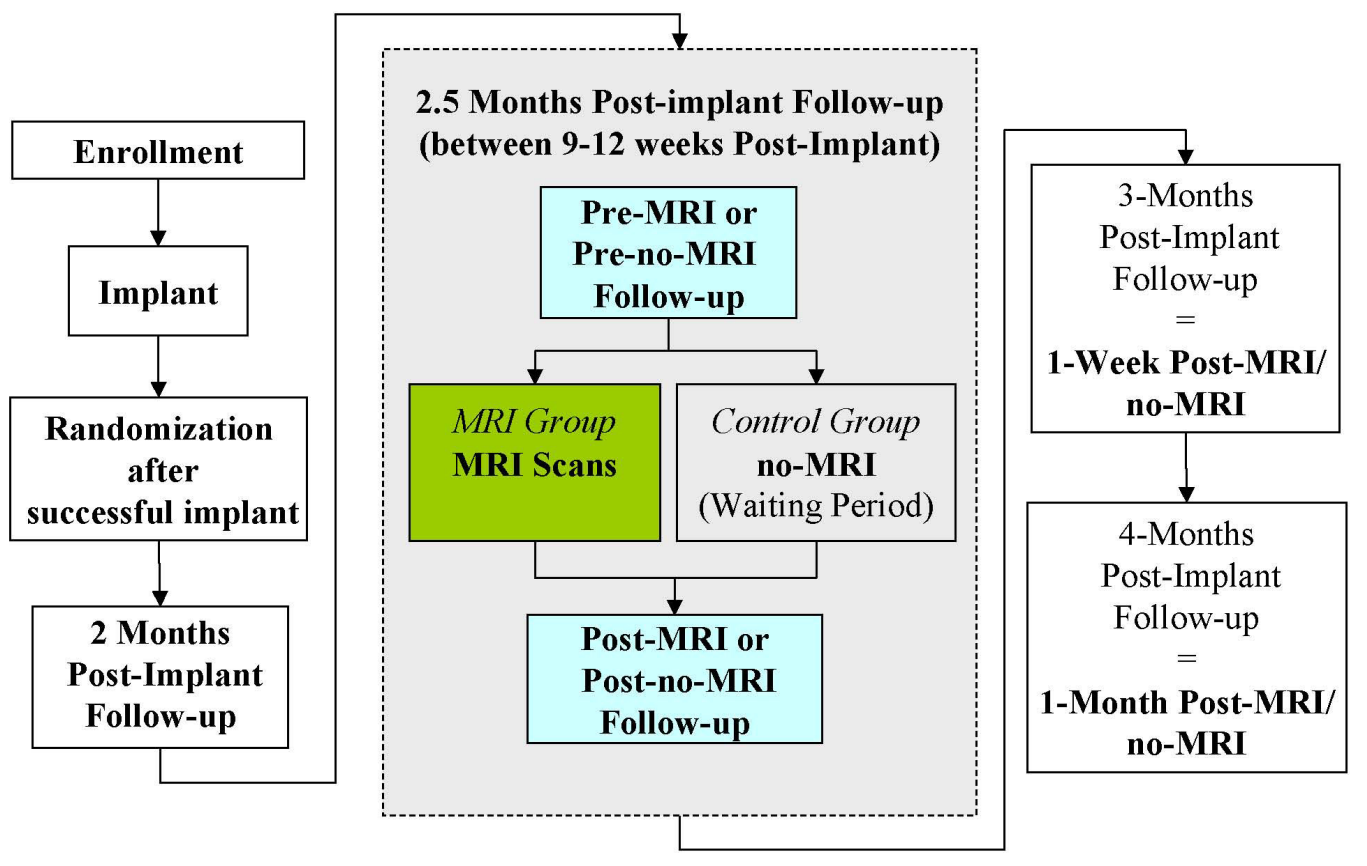
Study visit flowchart.

Patients were originally planned to be randomized in a 1:1 ratio, but later during the trial the ratio was modified to a 2:1 ratio to undergo an MRI scan (MRI group) or not to undergo an MRI scan (control group) after a successful implantation of an EnRhythm MRI SureScan pacing system (Figure 1). It is anticipated that more than 60% of the implanted patients will be randomized in a 1:1 order and the remainder in a 2:1 order. The ratio was adjusted so as to meet regulatory requirements for the minimum number of patients scanned.

### MRI scanning

Patients randomized to receive MR scanning undergo imaging at 9–12 weeks after implantation. Fourteen clinically relevant brain and lumbar spine MR scans are performed in each patient of the MRI group (total MRI investigation time of approximately 45 minutes, total active MRI scan time of approximately 30 minutes). According to the labelling of the MRI SureScan Pacing system, all MRI studies are performed at a 1.5 Tesla system, the slew rate ("gradient dB/dt") of the gradient system of each individual MR sequence is restricted to 200 T/m/s and the radiofrequency exposure of each individual MR sequence is restricted to a specific absorption rate (SAR) of 2 W/kg body weight or less. Furthermore, labelling requires that the isocenter of the RF coil falls above the first cervical vertebral body (C1) or below the twelfth thoracic vertebral body (T12). The sequences to be used were carefully chosen by the study's Scan Advisory Committee to be clinically applicable and similar between different commercially available 1.5 T closed bore MRI scanners (manufacturers GE, Siemens, Philips) and to include sequences utilizing high gradient slew rates and/or producing high SAR close to the labelling limits of the pacemaker system. Some of the MR scan protocols with already high gradient slew rates were modified to maximize the gradient exposure up to the system limit or if possible to 200 T/m/s. In addition, to maximize gradient exposure in the lumbar spine scans, the patient is placed in such a way (i.e., isocenter of the RF coil is at L1 and S1) that the highest induced voltages at the pacing system site are expected. At least one lumbar spine scan should achieve high SAR values. Validation of all scans is required by each center prior to executing the first study patient scan in order to ensure scan protocols for each particular site are comparable across sites.

At minimum, patients are continuously monitored via pulse oximetry during the scans. Verbal communication also takes place to assess or confirm any significant clinical changes. Voltage stimulation thresholds, sensing amplitudes and impedance, will be measured in both the atrium and the ventricle immediately before the MRI scans are started and immediately after the MRI scans are completed.

Although the clinical study includes a fixed set of brain and lumbar spine sequences, the MRI scanning requirements of the EnRhythm MRI pacing system makes it possible to perform diagnostic MRI scans of the anatomic region within C1 and T12 while placing the isocenter of the RF coil outside of this zone by changing certain MRI parameters.

### Objectives of the study

Two sets of objectives will be analyzed. The first will be analyzed after approximately 156 patients have been implanted with this new pacemaker system and followed for 4 months to provide data for Medtronic market release outside of the US and the second analysis is after approximately 470 patients have been implanted and are followed for 4 months after receiving this new pacemaker system for regulatory requirements purposes.

The first set of objectives related to the 156 patients implanted and followed-up is described below:

#### a) Primary Objectives

1. To assess if the pacing system-, MRI- and implantation procedure related complication rate from implantation until one month post-MRI is greater than 80%. A one-sided exact test with a 95% confidence interval is used for analysis.

An adverse event (AE) is pacing system-related if it results from the presence or performance of the system under investigation. An AE or adverse device effect is MRI-procedure related if it is caused by the interaction between the investigational pacing system and the MRI system that occurs during the MRI procedure and includes the time the patient is within the 5 Gauss line of the MRI system through the subject's 4 months follow-up (1 month post MRI). In addition, AEs occurring due to the subject's MRI programming will be considered MRI procedure-related. An AE is implant-procedure related if it occurs due to the implant procedure. An AE is classified as a complication if it results in invasive intervention or the termination of significant device function regardless of other treatments. Intravenous and intramuscular drug therapies are considered invasive treatments.

2. To compare the changes in atrial and ventricular voltage stimulation thresholds at 0.5 ms, before and one month after MRI/no-MRI, between the MRI and control groups. Changes in threshold are used to indicate potential myocardial thermal damage. The hypothesis will test whether the changes are statistically equivalent (Δ = 1 V) between both groups. A two-sided confidence interval will be used for analysis.

3. To compare the differences in atrial and ventricular sensing amplitudes one month after MRI/no-MRI between the MRI and control groups, making a statistical comparison of the effect of MRI on these parameters. The hypothesis will test whether the sense amplitudes are statistically equivalent (Δ = 1.7 mV atrium, 5.0 mV ventricle). A two-sided confidence interval will be used for analysis.

All 3 primary objectives need to be met in order to pass this first analysis of the study.

#### b) Secondary Objectives

All secondary objectives and additional analyses are analyzed using descriptive statistics but are not statistically evaluated.

1. Confirm that labelling instructions for completing the MRI scans were followed to ensure patient safety. Occurrence of system-related adverse device effects due to insufficiencies of the labelling or inadequacies of the labelling are used.

2. Characterize occurrence of sustained ventricular arrhythmias and asystole seen during MRI scans.

3. Characterize all implant procedure-, pacing system- and MRI procedure-related complications and observations to four months post-implant.

4. Summarize atrial and ventricular lead impedance up to four months post-implant.

5. Characterize the lead handling of the CapSureFix MRI lead model 5086 MRI in relation to the commercially available model 5076. A seven-point questionnaire is used.

6. Characterize four months pacing threshold and sense amplitude of the MRI group and control group in relation to the commercially available lead Model 5076.

#### c) Additional Analysis

**Figure 2:**
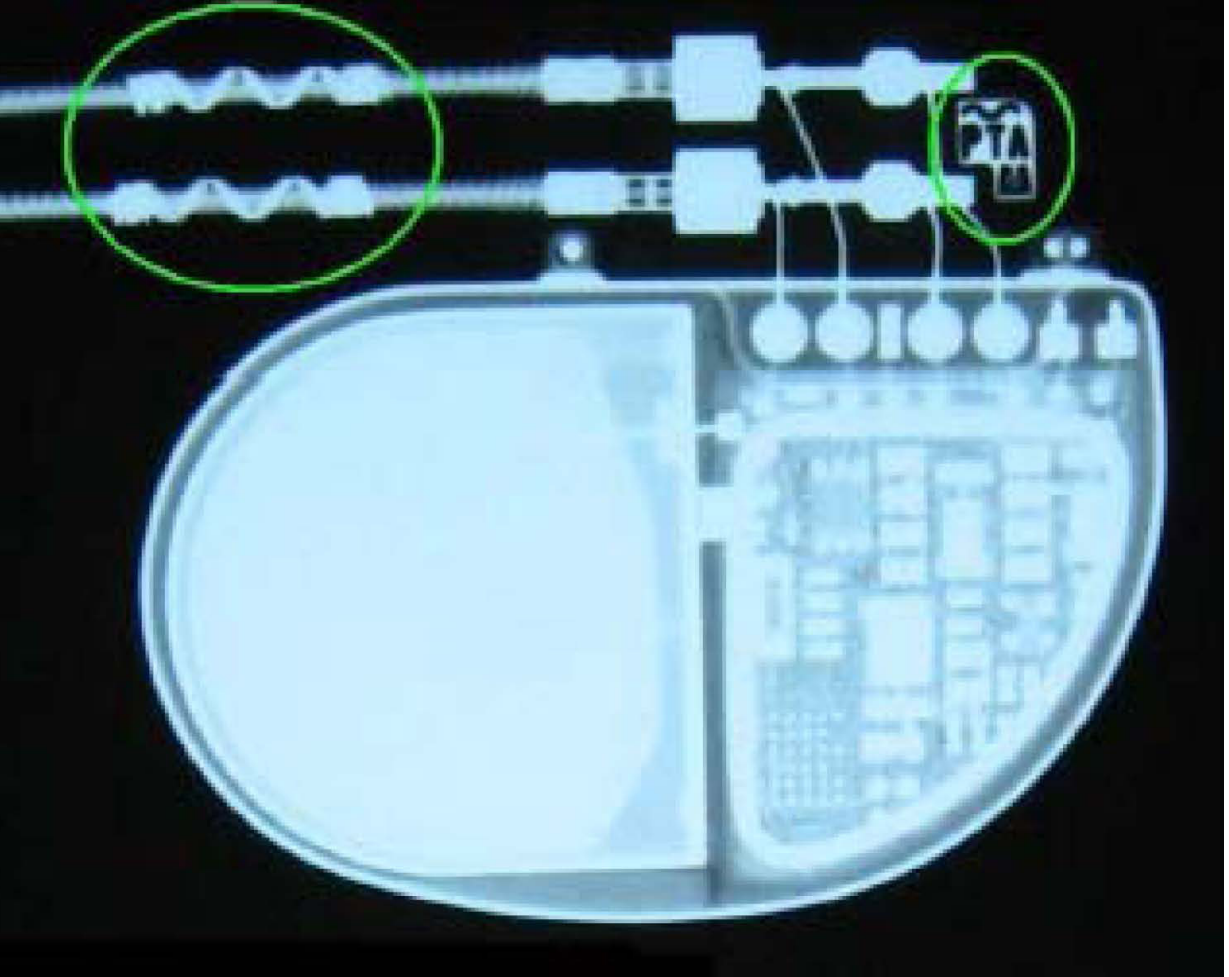
Radiopaque labels on lead and IPG to identify that patient has an MRI-conditional system implanted.

1. Demonstrate that the EnRhythm MRI SureScan Pacing System (both generator and leads) can be identified as MRI-labelled with X-ray (Figure 2). A seven-point questionnaire is used.

The second analysis after 470 patients have been implanted and followed-up for four months contains the following objectives:

#### a) Primary Objectives

1. To assess if the MRI-related complication-free rate in the month following MRI is greater than 90%. A one-sided exact test with a 97.5% confidence interval is used for analysis.

2. To compare the changes in a) atrial and b) ventricular voltage thresholds at 0.5 ms before and one month after MRI/no-MRI between the MRI and control groups. The hypothesis will test whether the proportions of patients (p_1_and p_2_) that experience an acceptable threshold increase less or equal to 0.5 V are equivalent (Δ = 10%, p_1 _and p_2 _= 96%) between the two groups. The objective will be analyzed using a two-sample 97.5% confidence interval, with p-values from the Farrington-Manning test for equivalence of proportions.

3. To compare the changes in a) atrial and b) ventricular sense amplitudes before and one month after MRI/no-MRI between the MRI and control groups. The hypothesis will test whether the proportions of patients (p_1 _and p_2_) who experience a sense amplitude decrease less than 50% and whose sense amplitudes remain above an clinically acceptable minimum (1.5 mV for atrium and 5 mV for the ventricle) at one month post-MRI/no-MRI are equivalent (Δ = 10%, p_1 _and p_2 _= 93%) between the two groups. The objective will be analyzed using a two-sample 97.5% confidence interval, with p-values from the Farrington-Manning test for equivalence of proportions.

All 3 primary objectives of this second set need to be met in order to pass the study.

#### b) Secondary Objectives

In addition to the six secondary objectives listed above for the first analysis, a further objective will characterize all system-related complications between the implant procedure and the four month follow-up visit to confirm that the complication-free rate is greater than 80%. The objective is analyzed using a one-sided exact test with a 95% confidence interval.

The secondary objectives for system-related complications, lead handling, and lead performance are evaluated under the "fixed-sequence method" in order to preserve the overall type one error of these objectives for regulatory purposes. The three objectives are tested sequentially with formal statistical tests. Lead handling is analyzed using a 95% confidence interval to assess whether the differences in overall lead handling characteristics are statistically equivalent between the EnRhythm MRI cohort and the 5076 historical comparison group (Δ = 1.5 units). Lead performance is analyzed using 95% confidence intervals to analyze whether the differences in capture and sensing are statistically equivalent between the EnRhythm MRI cohort and the 5076 historical comparison group (Δ = 0.5 V for capture, 0.9 mV for atrial sensing, and 2.5 mV for ventricular sensing).

#### c) Additional analyses

In addition to the additional analysis listed above for the first analysis, a further analysis will summarize aberrant or undesirable behaviour of the MRI SureScan programming mode, for which information is collected pertaining to its use and performance.

Furthermore, a summary of whether safeguards and procedures were followed at the time of MRI scans will be provided. Information is collected pertaining to whether safeguards and procedures for the preparation, programming, and monitoring around the time of the MRI scans were followed for the cardiology and radiology teams.

### Sample size and data use for analysis

Sample size calculations for both sets of objectives exclude attrition and provide at least 80% power with a type one error rate (alpha) of 0.05 (first set) or 0.025 (second set). No alpha adjustment was necessary for the two analyses, since the objectives are different and both sets will each be analyzed only once. Sample sizes were calculated separately for each primary objective and the largest requirements (62 patients per group for the first set, 122 per group for the second set) were further increased to 156 and 470 total patients, respectively, for consideration of estimated study attrition and added regulatory requirements.

Pre-specified analysis exclusion criteria were listed in the investigational plan and include the following:

• Data from visits outside the 1 month post-MRI visit window are excluded from the primary analyses of capture and sensing objectives, but are included in a secondary sensitivity analysis of these objectives.

• Data from patients whose MRI scans were not performed according to protocol requirements are excluded from the analyses of capture, sensing, MRI-related complications, labeling instructions, occurrence of arrhythmias, and lead impedance.

• Data from patients in atrial arrhythmias at the time of electrical data collection are excluded from analyses of atrial capture and sensing objectives at affected visits.

• Data from patients with unresolved lead dislodgments are excluded from analyses of capture, sensing, and impedance objectives.

• Data from patients with a pacing threshold difference exceeding 0.5 V prior to the MRI visit, which is indicative of an abnormal lead/tissue interface, are excluded from analyses of capture and sensing objectives.

• Pacing capture data lacking required threshold strip documentation or not measured at 0.5 ms are excluded from the analysis of the capture threshold objective.

• Patients unable to capture will be assigned a capture threshold value of 6 V at affected visits for the purpose of calculating descriptive statistics.

### Independent advisory committees

The Investigator will be asked to assess the relationship of each adverse event to the pacing system, implant procedure and/or the MRI scan. An Adverse Event Advisory Committee (AEAC) will review the classifications of all adverse device effects, deaths, technical observations and adverse events at regular intervals.

A Scan Advisory Committee developed similar MRI scan protocols for the different MRI machines from the three manufacturers and assisted in the validation of the individual MRI scans. Furthermore, this committee will regularly review the performed MRI scans and determine if the scan data acquired will be included in the analysis.

The trial is supervised by an independent data monitoring committee (DMC), consisting of one statistician, one radiologist and one cardiologist, to review safety data as well as to review periodically the efficacy data.

### Ethical aspects

The study will be conducted according to the investigational plan, the Declaration of Helsinki concerning medical research and in accordance with local laws and regulations of countries where patients are enrolled. Each center needs Institutional Review Board/Medical Ethics Committee approval of the study protocol and written patient informed consent prior to enrolment. Patients may withdraw from the study at any time without giving reasons and without jeopardizing their further treatment. The investigator may also withdraw patients if this is in their best interests.

## Discussion

This study has been designed to confirm the safety and efficacy of a new pacing system in the MRI environment.

There is much published work on the effects of MRI scans on patients with implanted devices and recommendations for minimizing these hazards [4-6,8-15]. The inference of these reports indicates that dedicated pacing systems are needed to avoid complications including arrhythmias, inhibition of pacing output and triggered stimulations, and RF-related heating of the pacing leads with potential thermal damage at the electrode/tissue interface, although there remains some controversy over this need [3]. Systems designed for safe use in the MR environment must be considered in the light of a rapidly increasing number of MR studies being performed worldwide in all types of patient. Implanted device patients at present are clearly clinically disadvantaged by being unable safely to undergo MRI in many conditions that they may develop.

A new system has been designed to address the safety issues of MRI scanning in patients with implanted devices. This requires careful clinical assessment, which is the object of this trial using a randomization technique to those who will be scanned and a control group who will not.

In the future, it is hoped, assuming this trial to be successful, that MRI-compatibility may be extended to more complex devices such as those that deliver cardiac resynchronization therapy and implantable cardioverter defibrillators.

## Abbreviations

ACC: American College of Cardiology; AHA: American Heart Association; AE: Adverse Event; AEAC: Adverse Event Advisory Committee; C1: Cervical Vertebra 1; DMC: Data Monitoring Committee; HRS: Heart Rhythm Society; ICD: Implantable Cardioverter Defibrillator; IPG: Implantable Pulse Generator; MR: Magnetic Resonance; MRI: Magnetic Resonance Imaging; T12: Thoracic Vertebra 12; US: United States; SAR: Specific Absorption Rate.

## Competing interests

RS, EKl, DB, RLr and TS are consultants to Medtronic. BW is a consultant, member of a scientific advisory board, receives royalties and has other research support from Medtronic. IJ and MH are both employees of Medtronic.

## Authors' contributions

RS and IJ wrote the paper and submitted it to the other authors who returned comments and revisions. All authors have contributed to the design of the study. Medtronic is the sponsor of this study and the study was designed in cooperation with all authors.
